# Hot Deformation Behavior of the 25CrMo4 Steel Using a Modified Arrhenius Model

**DOI:** 10.3390/ma15082820

**Published:** 2022-04-12

**Authors:** Hongtu Xu, Tiantai Tian, Jiahao Zhang, Liqun Niu, Hongbin Zhu, Xingtao Wang, Qi Zhang

**Affiliations:** 1 School of Mechanical Engineering, Xi’an Jiaotong University, Xi’an 710049, China; xuhongtu1996@126.com (H.X.); tiantai319@stu.xjtu.edu.cn (T.T.); hao18791506037@stu.xjtu.edu.cn (J.Z.); niuliqun111@126.com (L.N.); 2 CRRC Industrial Academy Co., Ltd., Beijing 100070, China; zhuhongbin@crrcgc.cc (H.Z.); wangxingtao@crrcgc.cc (X.W.)

**Keywords:** 25CrMo4 steel, constitutive model, microstructure evolution, hot deformation

## Abstract

25CrMo4 steel is widely used in the manufacturing of high-speed train axles due to its excellent mechanical properties. The purpose of this study is to develop an accurate modified constitutive model to describe the hot deformation behavior of the steel. Isothermal compression experiments were performed at different strain rates (0.01, 0.1, 0.5, and 1 s^−1^) and different temperatures (950, 1000, 1050, and 1100 °C) using a Gleeble-3800 thermal simulator. The microstructure after hot deformation was observed by the electron backscatter diffraction (EBSD), and the effects of temperature and strain rate were analyzed. The results showed that the coupling effect of temperature and strain rate on the dislocation density led to the change in the shape of the true stress–strain curve and that dynamic recovery (DRV) and dynamic recrystallization (DRX) caused the macroscopic softening phenomenon, with DRX being the main mechanism. Based on the true stress–strain curves, the strain-compensated Arrhenius constitutive model was calibrated. To improve prediction ability, a modified Arrhenius constitutive model was proposed, in which the temperature and strain rate coupling correction functions were incorporated. The original, modified Arrhenius models were evaluated according to the absolute relative error (ARE), the average absolute relative error (AARE), and the correlation coefficient (R^2^). Compared with the original model, the modified Arrhenius model has a higher prediction accuracy, with the ARE value mostly below 4%, the AARE value of 1.91%, and the R^2^ value of 0.9958.

## 1. Introduction

Low carbon alloy steel 25CrMo4 is widely used in the manufacturing of high-speed train axles because of its excellent mechanical properties, such as high strength, good toughness, good hardenability, and high impact load resistance [1,2]. Because of strict size restrictions and operational regulations, high-speed train axles require periodic refurbishment or replacement, and every year, rail operators around the world replace thousands of axles. Blank parts for high-speed train axles are usually produced by hot deformation processes, of which heavy forging and free forging are the main ones [3]. Due to the world’s resource shortage and the increasing pressure to save energy and reduce emissions in the future, it is crucial to shortening the hot processing process. It is well known that finite element simulation (FES) is an effective method for process design and optimization, and the constitutive model determines the accuracy of the FES [4,5]. Therefore, it is very necessary to develop an accurate constitutive model to predict the hot deformation behavior of 25CrMo4 steel.

At present, there have been some reports on studying the hot deformation behavior of 25CrMo4 steel. Xu et al. [6] studied the compressive deformation behavior of 25CrMo4 steel at 950–1150 °C, calibrated the Arrhenius model, and determined the deformation activation energy, which was found to be 309.5 KJ/mol. Huo et al. [7] investigated the hot deformation behavior of 25CrMo4 steel and the effect of process parameters such as temperature, strain rate, and strain on the evolution of microstructure by metallographic analysis. Zhou et al. [8,9] studied the static recrystallization and dynamic recrystallization behavior of 25CrMo4 steel and developed the kinetic models, and the experimental and predicted results were in good agreement. Liu et al. [10] investigated the deformation behavior and microstructure of 25CrMo4 steel by hot compression experiments, calibrated the Arrhenius model and hot processing map and determined the appropriate hot deformation process parameters with deformation temperatures of 1050–1200 °C and strain rates of 0.01 to 0.14 s^−1^. Although the original Arrhenius model was calibrated and some results and contributions were achieved, the prediction accuracy of the constitutive model needs to be further improved in order to shorten the hot processing process as the pressure of energy saving and emission reduction increases.

Generally, there are three types of constitutive models: physical-based constitutive model, neural network-based constitutive model, and phenomenological constitutive model [11]. The physics-based constitutive model [12,13,14] can relate material properties to their microstructure and deformation mechanisms, but a large number of internal variables and the complexity of calculations make it unsuitable for FES. Neural network-based constitutive models [15,16,17] usually have high prediction accuracy but are difficult to combine with FES and lack practicality. The phenomenological constitutive model does not consider deformation mechanisms and microstructural changes define the flow stress as a function of strain, strain rate, and temperature and uses experimental data to calibrate the model. This model has fewer material parameters, simpler experimental and calibrated schemes, and is widely used in FES [4,5]. So far, many phenomenological models are used for the prediction of the hot deformation behavior of metals, such as the Johnson–Cook (JC) model [18,19], the Norton–Hoff model [20], the Zerilli–Armstrong model [21], and the Arrhenius model. The Arrhenius model contains the thermal activation energy Q, which has a specific physical meaning and a relationship with the material microscopic deformation mechanism and has been widely used. Lin et al. [22] modified the Arrhenius model to describe the flow behaviors of 42CrMo steel by the compensation of strain with an error of 4.36% between predicted stresses and experimental results. The strain-compensated Arrhenius model has been successfully used to predict the hot deformation behavior of other alloys, such as titanium alloys [23], magnesium alloys [24], aluminum alloys [25], and super alloys [26], and its accuracy can be further improved. Lin et al. [22] and Mandal et al. [27] corrected the model by adding a strain rate index to the Zener–Hollomon parameter; however, this method requires that the predicted and experimental results agree well at a strain rate of 1 s^−1^. Wang et al. [28] modified the model by replacing the true strain rate with the effective strain rate. This method calculated the effective strain rate from the material constants calibrated at the true strain rate and then substituted the effective strain rate into the model. Although the prediction accuracy of the model was improved, the calculation was complicated and did not consider the effect of microstructure. Chen et al. [29] introduced a polynomial function of temperature and strain rate to modify the model, and the error was further reduced, but the computational effort was huge due to the introduction of eight parameters. Yi et al. [30] modified the strain rate index in the Zener–Hollomon parameter by using a locally weighted smoothed quadratic regression method to obtain the relationship between the index, temperature, and strain rate. The modified model predicted well under most conditions, but at 920 °C and a strain rate of 0.28 s^−1^, there was a significant deviation in the predicted data. The reasons affecting the accuracy of the model may be the inherent limitations of the nonlinear equations and the evolution of the microstructure during hot deformation [5]. Therefore, in order to reduce carbon emissions and the hot processing process, it is needed to analyze the evolution of microstructure during the hot deformation for the 25CrMo4 steel and further modify the constitutive model.

In this study, the isothermal compressive deformation behavior of the 25CrMo4 steel at 950–1100 °C and strain rate of 0.01–1 s^−1^ was investigated. The microstructures of the specimens at different temperatures and strain rates were observed by the electron backscatter diffraction (EBSD), and the hot deformation mechanism was analyzed. The strain-compensated Arrhenius model was calibrated, and a modified Arrhenius model considering the coupling of temperature and strain rate was proposed based on the microstructure evolution. Compared with other modified Arrhenius models, the model introduced fewer parameters, was relatively simple to calculate, and had a higher accuracy.

## 2. Materials and Methods

**Figure 1:**
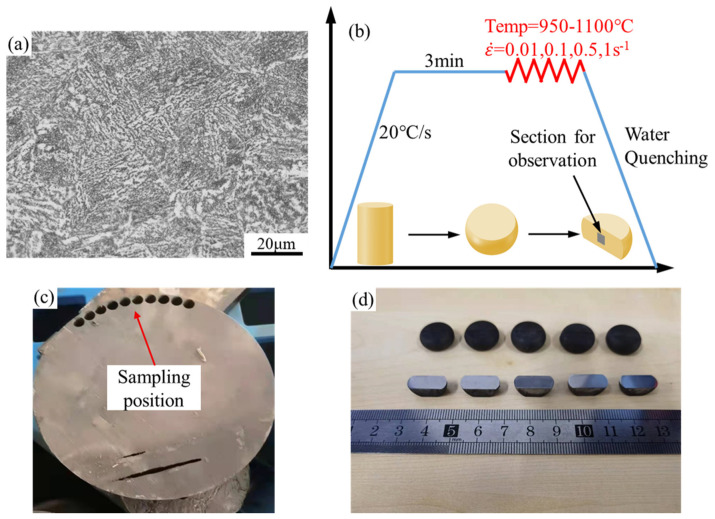
(**a**) Microstructure of the as-received specimen; (**b**) Experimental procedure of compression tests; (**c**) Sampling position; (**d**) Hot compressed specimens and polished section.

The material used in this study was the 25CrMo4 steel supplied by the CRRC Industrial Academy Co., Ltd. The original microstructure of the steel is shown in Figure 1a, and the chemical composition, as shown in Table 1, was provided by the manufacturer. Compressed specimens were prepared in accordance with ASTM E209 [31]. Cylindrical specimens were machined with a diameter of 10 mm and a height of 15 mm. Isothermal compression tests were performed on Gleeble-3800 thermal simulator at four different temperatures (950, 1000, 1050, and 1100 °C) under different strain rates (0.01, 0.1, 0.5, and 1 s^−1^). As shown in Figure 1b, the specimens were heated to compression temperatures at a heating rate of 20 °C/s and held for 3 min, and the temperature of the sample was precisely controlled by a thermocouple. The specimens were compressed to a true strain of 0.8 and then quenched by water immediately.

Figure 1c,d shows the sampling position and the compressed specimens, respectively. The hot deformed specimens were sectioned along the longitudinal compression axis. Then the sections were mechanically polished with sandpaper and polished by Ion thinner (Leica EM RES102 from German) at 2 kv for 4 h. EBSD was measured by scanning electron microscope (Hitachi SU3500 from Japan) with a scan step size of 0.35 μm. HKL Channel5 software was used for the experimental data analysis.

## 3. Results and Discussion

### 3.1. Flow Stress Behavior

**Figure 2:**
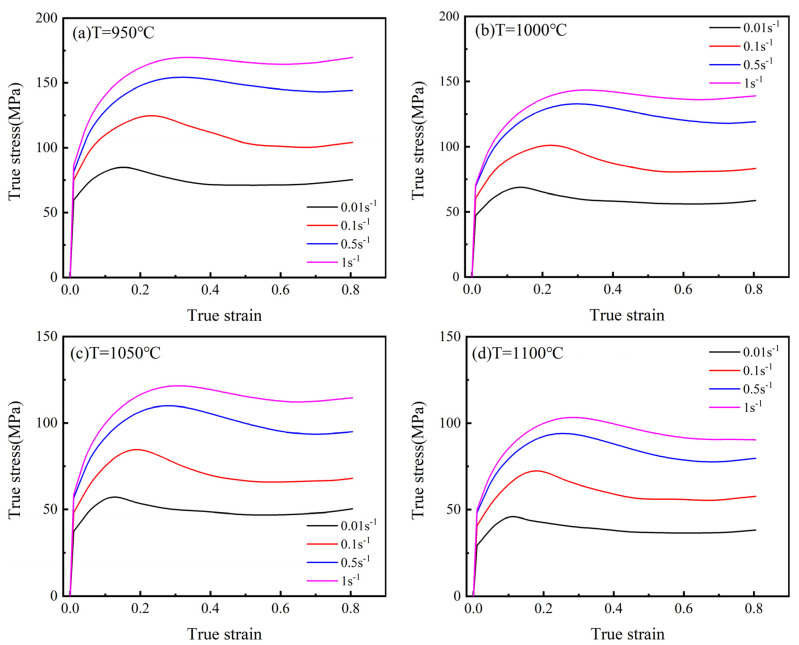

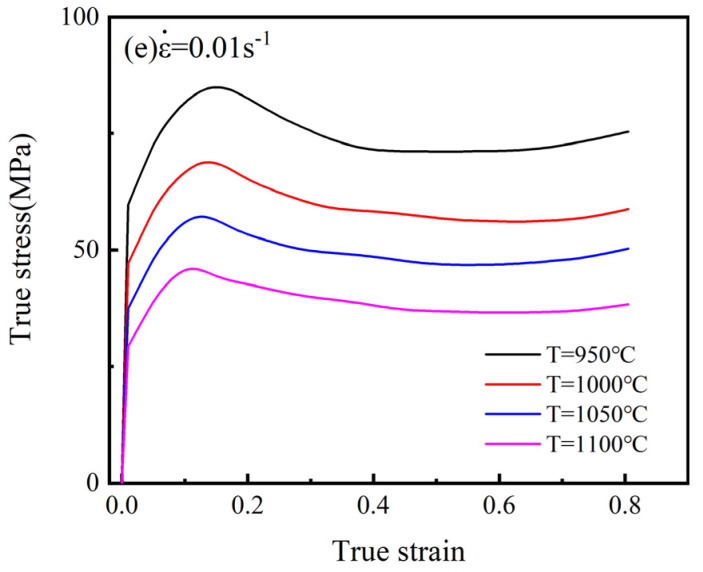
True stress–strain curves at different temperatures and strain rates. (**a**) T = 950 °C; (b) T=1000 °C; (**c**) T = 1100 °C; (**d**) T = 1100 °C; (**e**) $\dot{\varepsilon }$ = 0.01 s^−1^.

The flow curves of the 25CrMo4 steel at deformation temperatures of 950–1100 °C and strain rates of 0.01–1.0 s^−1^ are presented in Figure 2. In the early stage of deformation, the flow stress increases rapidly with increasing strain, which is mainly due to the work hardening (WH) caused by the generation and proliferation of dislocations [32], and the growth rate of the stress becomes smaller due to dynamic recovery (DRV). When the stress reaches its peak, dynamic recrystallization (DRX) occurs, consuming a large number of dislocations, so the flow stress gradually decreases. However, the hot deformation behavior at 950–1000 °C with a strain rate of 1 s^−1^ shows a trend of WH, which may be due to the incomplete recrystallization phenomenon resulting in the insufficient recovery of WH at lower temperatures and higher rates [33]. It is noteworthy that as the strain rate increases and the temperature decreases, the peak stress and critical strain gradually increase.

### 3.2. Microstructure Evolution

**Figure 3:**
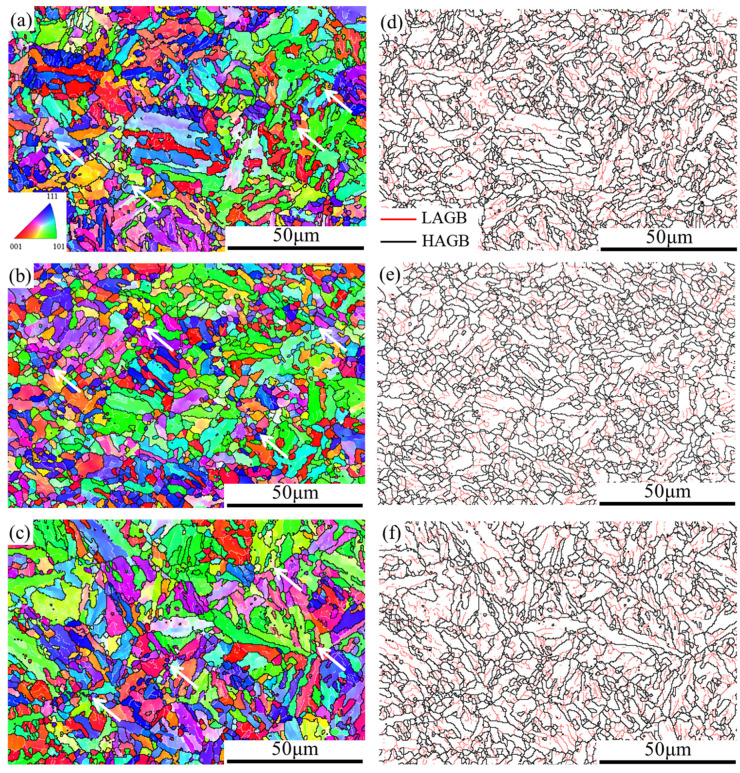
The EBSD maps and misorientation of the grain boundaries at varying compression conditions. (**a**,**d**) T = 1000 °C, $\dot{\varepsilon }$ = 0.1 s^−1^; (**b**,**e**) T = 1100 °C, $\dot{\varepsilon \;}$ = 0.1 s^−1^; (**c**,**f**) T = 1000 °C, $\dot{\varepsilon \;}$ = 1 s^−1^.

**Figure 4:**
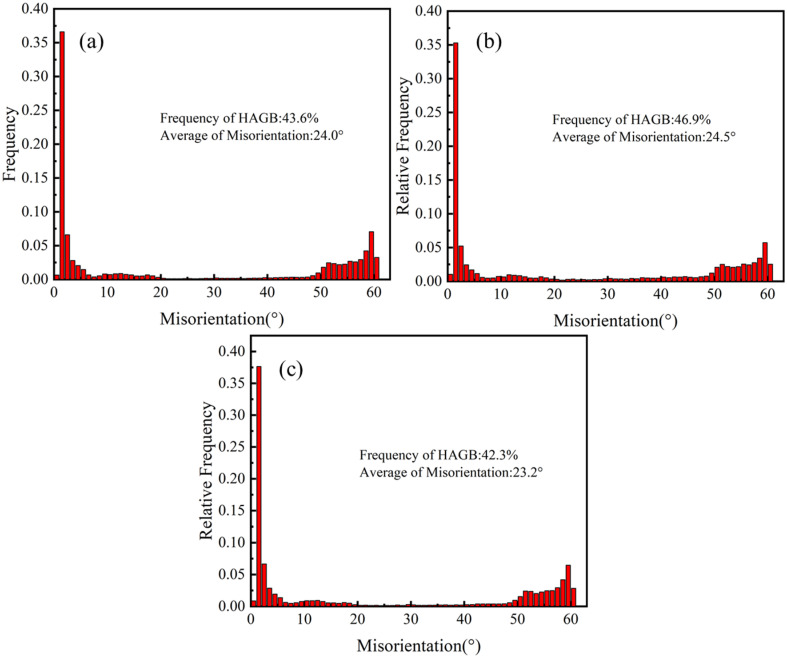
The misorientation of the grain boundaries at varying compression conditions. (**a**) T = 1000 °C, $\dot{\varepsilon \;}$ = 0.1 s^−1^; (**b**) T = 1100 °C, $\dot{\varepsilon }$ = 0.1 s^−1^; (**c**) T = 1000 °C, $\dot{\varepsilon \;}$ = 1 s^−1^.

In order to study the microstructure evolution of the 25CrMo4 steel during hot compression, the microstructure of compressed specimens with a true strain of 0.8 and different deformation parameters was characterized. Figure 3 and Figure 4 show the EBSD maps and misorientation of the grain boundaries under different strain rates and deformation temperatures, respectively, where the red lines represent the low angle grain boundaries (LAGB) with the misorientation angle between 2° and 15°, and the black lines represent the high angle grain boundaries (HAGB) with the misorientation angle greater than 15°. It can be seen that DRX occurs in all deformation conditions, and fine recrystallized grains are produced at grain boundaries and subgrain boundaries, as shown by the white arrows.

As the temperature increases, the number of LAGB decreases, the number of HAGB increases, and fine new grains are formed inside some grains. Since dislocation movement is a thermally activated process, high deformation temperature can provide enough energy for dislocation slip, creep, and cross-slip, which facilitates the long-term movement of dislocations [34]. During migration, dislocations accumulate continuously and form dislocation walls and subgrain boundaries after reaching the grain boundaries. When the dislocation density reaches a critical value, LAGB transforms into HAGB, and DRX occurs, leading to the formation of fine new grains. At the same time, the activation of dislocation movement will also accelerate the annihilation of dislocations [35], which eventually leads to a decrease in the LAGB ratio and an increase in the average misorientation. As shown in Figure 4a,b, the frequency of HAGB increases from 43.6% to 46.9%, and the average misorientation angle increases from 24° to 24.5° when the temperature increases from 1000 °C to 1100 °C. The average misorientation angle increases slightly and almost tends to be constant, indicating that the softening mechanism is basically stable under such conditions. As the strain rate increases, the number of LAGB increases, which shortens the time of dislocation movement and reduces the occurrence of DRV and DRX; however, the microstructures of Figure 3a,c are similar, indicating that their deformation mechanisms are basically the same. As shown in Figure 4a,c, the frequency of HAGB decreased from 43.6% to 42.3%, and the average misorientation angle decreased from 24% to 23.2% with an increasing strain rate.

**Figure 5:**
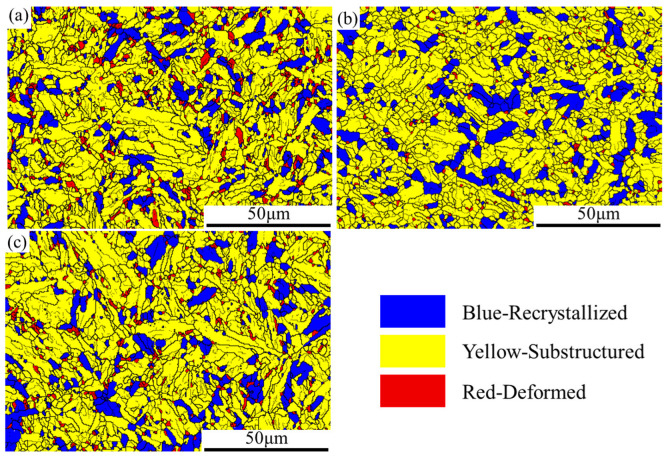
The EBSD maps at different compression conditions. (**a**) T = 1000 °C, $\dot{\varepsilon \;}$ = 0.1 s^−1^; (**b**) T = 1100 °C, $\dot{\varepsilon \;}$ = 0.1 s^−1^; (**c**) T = 1000 °C, $\dot{\varepsilon \;}$ = 1 s^−1^.

For further analysis of the DRX characteristics and microstructure evolution, the results of DRX are expressed by the grain orientation difference, as shown in Figure 5. The grains with an orientation difference of less than 2° are defined as recrystallized, greater than 2° but less than 15° is defined as substructured, and more than 15° is defined as deformed. When the strain rate is 0.1 s^−1,^ and the deformation temperatures are 1000 °C and 1100 °C, the DRX fractions are 17.7% and 19.8%, as shown in Figure 5a,b, respectively. As the temperature increases, the softening effect gradually dominates, and when the temperature rises to 1100 °C, a clear trend of decreasing flow stress can be observed, as shown in Figure 2d, even at the strain rate of 1 s^−1^. When the deformation temperature is 1000 °C, and the strain rates are 0.1 and 1 s^−1^, the DRX fractions were 17.7% and 17.1%, as shown in Figure 5a,c, respectively. With the increase in strain rate, less time is supplied to the DRX and DRV of grains during deformation, and the DRX fraction gradually decreases while the dislocation density increases. When the strain rate is 1 s^−1^, the WH effect exceeds the softening effect, so the hot deformation behavior shows a trend of WH, and the trend is more obvious at 950 °C.

**Figure 6:**
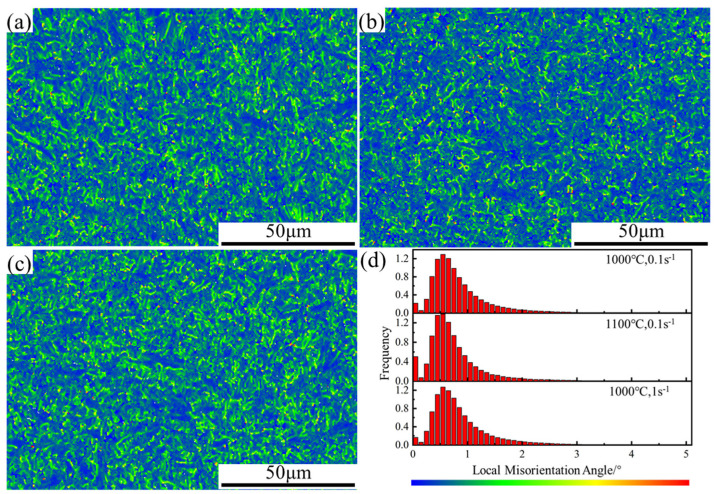
Distribution maps of local misorientation at different compression conditions. (**a**) T = 1000 °C, $\dot{\varepsilon \;}$ = 0.1 s^−1^; (**b**) T = 1100 °C, $\dot{\varepsilon \;}$ = 0.1 s^−1^; (**c**) T = 1000 °C, $\dot{\varepsilon \;}$ = 1 s^−1^.

The local misorientation maps are shown in Figure 6, while higher degrees of misorientation show up as a brighter green color. The average local misorientation angles in Figure 6a–c are 0.83°, 0.76°, and 0.85°, respectively. Both an increase in temperature and a decrease in strain rate lead to a decrease in the local misorientation angle, which indicates that the dislocation density decreases with an increase in temperature and a decrease in strain rate. The reason is that the increase in temperature can provide enough energy for dislocation movement, which intensifies the accumulation and annihilation of dislocations, and there is enough time to absorb dislocations at lower low strain rates. During hot deformation, dislocations propagate and accumulate due to deformation. On the other hand, there are also dislocation counteracting processes driven by thermal activation, subgrain formation, and combination [34]. Early in the deformation, DRV and WH occur simultaneously, and it is difficult for DRV to counteract both the proliferation and accumulation of dislocations. When dislocations accumulate to a certain extent, LAGB transforms into HAGB, and DRX occurs, eliminating a large number of dislocations, which leads to a decrease in flow stress and peak stresses appear on the stress–strain curve. When the balance between nucleation and growth of recrystallized grains is reached, the stress–strain curve tends to stabilize. Higher temperatures increase the rate of grain boundary migration, intensify the annihilation of dislocations, and reduce the degree of WH, leading to a decrease in the flow stress and critical strain. The increase in strain rate decreases the rate of grain boundary migration, increases the dislocation density, and ultimately increases the flow stress and critical strain. The different peak stresses and critical strains at different temperatures and strain rates indicate that the coupling effect of temperature and strain rate on the dislocation density is macroscopically manifested as a change in the shape of the stress–strain curve.

### 3.3. Constitutive Modeling

#### 3.3.1. The Arrhenius Model

The Arrhenius model is usually used to describe the relationship between deformation temperature, strain rate, and flow stress. The equation is as follows [36]:

(1) $$\dot{\varepsilon }=AF\left(\sigma \right)\exp\left(-\frac{Q}{\mathrm{RT}}\right)$$

where

(2) $$F\left(\sigma \right)=\{\begin{array}{c} \sigma^{n_{1}},\alpha \sigma <0.8\\ \exp\left(\beta \sigma \right),\alpha \sigma >1.2\\ \sinh{\left(\alpha \sigma \right)}^{n},\mathrm{for}\;\mathrm{all}\;\sigma \end{array}$$

where $\dot{\varepsilon }$ is the strain rate (s^−1^), σ is the true stress (MPa), Q is the thermal activation energy (KJ/mol), T is the deformation temperature (K), R is the molar gas constant (8.314 J/mol^−1^ K^−1^), A, β, $n_{1}$, and n are the material constants, $\alpha =\beta /n_{1}$.

Since the constitutive model does not account for the effect of strain on the parameters, polynomial fitting methods are considered to describe the relationships between the material parameters and strain. The relationships between  α*,* n*,* Q, and lnA can be fitted by strain:

(3) $$\left\{ \begin{array}{c} \alpha \varepsilon =\alpha_{0}+\alpha_{1}\varepsilon +\alpha_{2}\varepsilon^{2}+\alpha_{3}\varepsilon^{3}+\alpha_{4}\varepsilon^{4}+\alpha_{5}\varepsilon^{5}+\alpha_{6}\varepsilon^{6}\\ n\varepsilon =n_{0}+n_{1}\varepsilon +n_{2}\varepsilon^{2}+n_{3}\varepsilon^{3}+n_{4}\varepsilon^{4}+n_{5}\varepsilon^{5}+n_{6}\varepsilon^{6}\\ Q\varepsilon =Q_{0}+Q_{1}\varepsilon +Q_{2}\varepsilon^{2}+Q_{3}\varepsilon^{3}+Q_{4}\varepsilon^{4}+Q_{5}\varepsilon^{5}+Q_{6}\varepsilon^{6}\\ \ln A\varepsilon =\ln A_{0}+\ln A_{1}\varepsilon +\ln A_{2}\varepsilon^{2}+\ln A_{3}\varepsilon^{3}+\ln A_{4}\varepsilon^{4}+\ln A_{5}\varepsilon^{5}+\ln A_{6}\varepsilon^{6} \end{array}\right.$$

The coefficients of the Arrhenius model were determined by using the method in Appendix A, as shown in Table 2.

#### 3.3.2. The Modified Arrhenius Model

The strain-compensated Arrhenius model was developed in the previous study, and the strain rate and temperature are fitted linearly in the calculation without considering the coupling effect between them. Therefore, a modified Arrhenius model considering the coupling of temperature and strain rate is proposed. The equation is as follows:

(4) $$\dot{\varepsilon }e^{m\left(T\right)\dot{\varepsilon }}=AF\left(\sigma \right)\exp\left(-\frac{Q}{\mathrm{RT}}\right)$$

where $e^{m\left(T\right)\dot{\varepsilon }}$ represents the coupling of temperature and strain rate. The increase in temperature provides higher energy for dislocation motion, which is conducive to dislocation slip and creep on the one hand and intensifies dislocation annihilation on the other. Therefore, m may be expressed as the following equation:

(5) $$m=m_{1}T+\frac{m_{2}}{T}$$

Similarly, the relationships between $\;m_{1}$,$\;m_{2}$ can be fitted by strain:

(6) $$\{\begin{array}{c} m_{1}\varepsilon =m_{10}+m_{11}\varepsilon +m_{12}\varepsilon^{2}+m_{13}\varepsilon^{3}+m_{14}\varepsilon^{4}+m_{15}\varepsilon^{5}+m_{16}\varepsilon^{6}\\ m_{2}\varepsilon =m_{20}+m_{21}\varepsilon +m_{22}\varepsilon^{2}+m_{23}\varepsilon^{3}+m_{24}\varepsilon^{4}+m_{25}\varepsilon^{5}+m_{26}\varepsilon^{6} \end{array}$$

Taking the true strain of 0.4 as an example, the calculation process of the material parameters is as follows. The value of α has been calculated in Appendix A, i.e., α = 0.0116. For all the stress levels, Equation (4) can be represented as the following:

(7) $$\dot{\varepsilon }e^{\left(m_{1}T+\frac{m_{2}}{T}\right)\dot{\varepsilon }}=A\sinh{\left(\alpha \sigma \right)}^{n}\exp\left(-\frac{Q}{\mathrm{RT}}\right)$$

Taking the logarithm of both sides of the above Equation (7):

(8) $$\ln\left[\sinh\left(\alpha \sigma \right)\right]=\frac{\ln\left(\dot{\varepsilon }\right)}{n}+\frac{\left(m_{1}T+\frac{m_{2}}{T}\right)\dot{\varepsilon }}{n}+\frac{Q}{n\mathrm{RT}}-\frac{\ln A}{n}$$

**Figure 7:**
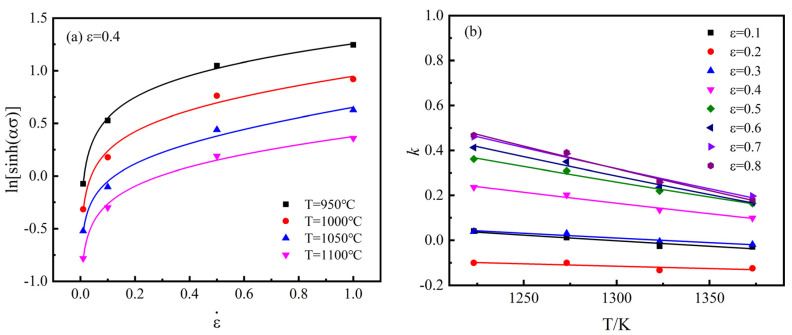
Fitting line (**a**) ln[sinh(ασ)] vs.$\;\dot{\varepsilon }$; (**b**) k vs. T.

When T is kept constant, the relationships between ln[sinh(ασ)] and $\dot{\varepsilon }$ are obtained, as shown in Figure 7a. The plots are fitted with the function: $\;y=\left(1/n\right)\ln\dot{\varepsilon }+k\dot{\varepsilon }+\mathrm{constant}$, k =$\;(m_{1}T+m_{2}/T)/n$, then taking the average value, n=4.2698 is obtained, The values of $m_{1}$ and $m_{2}$ are obtained from the fitting line k vs. T in Figure 7b, then taking the average values, $m_{1}=$−0.001761, $m_{2}=$3891.5457 are obtained.

**Figure 8:**
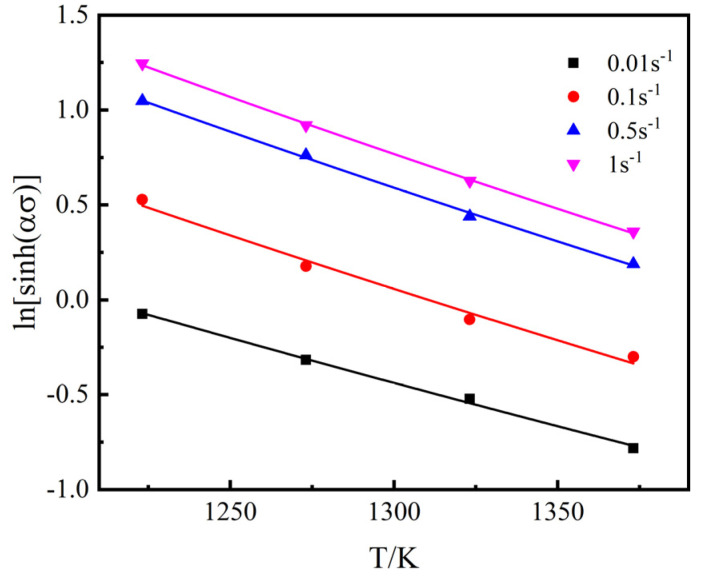
Fitting line, ln[sinh(ασ)] vs. T.

When $\dot{\varepsilon }$ is kept constant, the relationships between ln[sinh(ασ)] and T are obtained, as shown in Figure 8. The plots are fitted with the function:$\;y=k_{1}T+k_{2}\left(1/T\right)+\mathrm{constant}$, $k_{1}=m_{1}\dot{\varepsilon }/n$, $k_{2}=m_{2}\dot{\varepsilon }/n+Q/Rn$, then taking the average value, Q = 303.7526 KJ/mol is obtained.

In the modified Arrhenius model, Zener–Hollomon (Z) parameter can be expressed as:

(9) $$Z=\dot{\varepsilon }e^{\left(m_{1}T+\frac{m_{2}}{T}\right)\dot{\varepsilon }}\exp\left(\frac{Q}{\mathrm{RT}}\right)$$

Taking the logarithm of both sides of the above Equation (9):

(10) $$\mathrm{lnZ}=\ln\dot{\varepsilon }+\left(m_{1}T+\frac{m_{2}}{T}\right)\dot{\varepsilon }+\frac{Q}{\mathrm{RT}}=\ln A+n\ln\left[\sinh\left(\alpha \sigma \right)\right]$$

**Figure 9:**
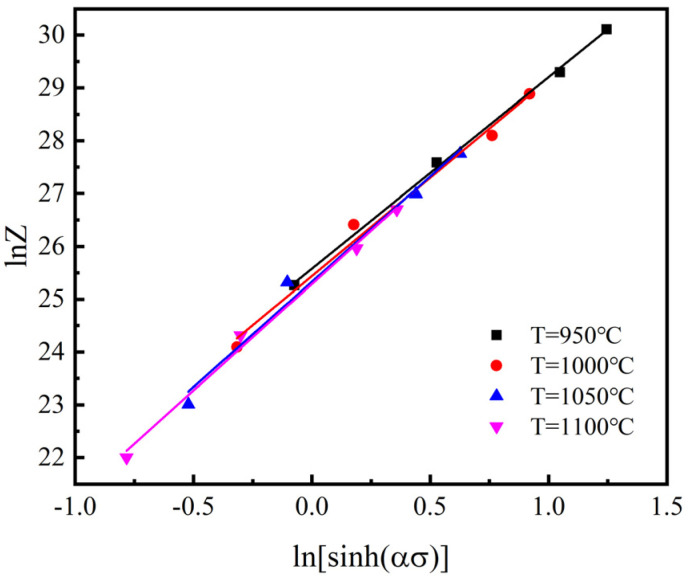
Fitting line, lnA by plotting lnZ vs. ln[sinh(ασ)].

The value of lnA is obtained from the intercept of the fitting line lnZ vs. ln[sinh(ασ)] shown in Figure 9, then taking the average value, lnA=25.4654 is obtained.

**Figure 10:**
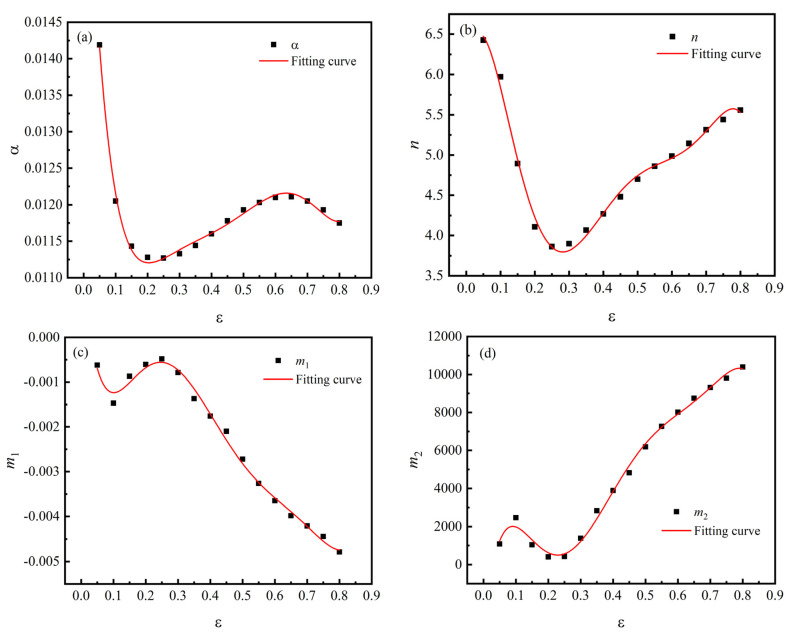

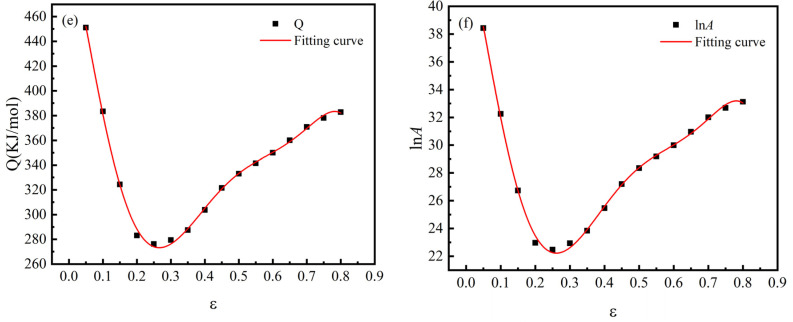
Variations of material constants with strain: (**a**) α; (**b**) n; (**c**) $m_{1}$; (**d**) $m_{2}$; (**e**) Q; (**f**) lnA.

According to the above method, the material constants are computed under different deformation strains within the range of 0.05–0.8 and the interval of 0.05. The relationships between material constants (α,$\;n,\;m_{1},\;m_{2},\;Q$, and lnA) and strain are shown in Figure 10, and the coefficients of the modified Arrhenius model are shown in Table 3.

## 4. Evaluation of Constitutive Models

**Figure 11:**
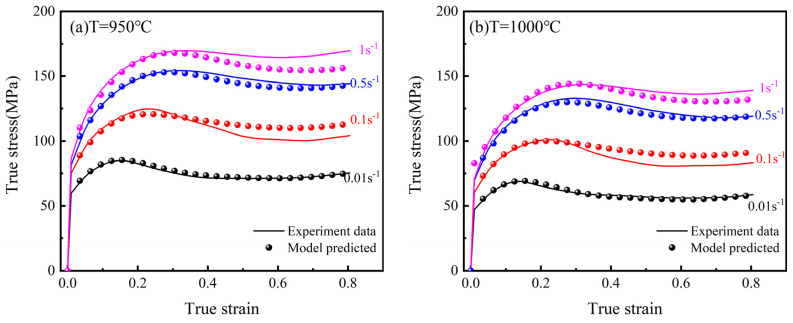

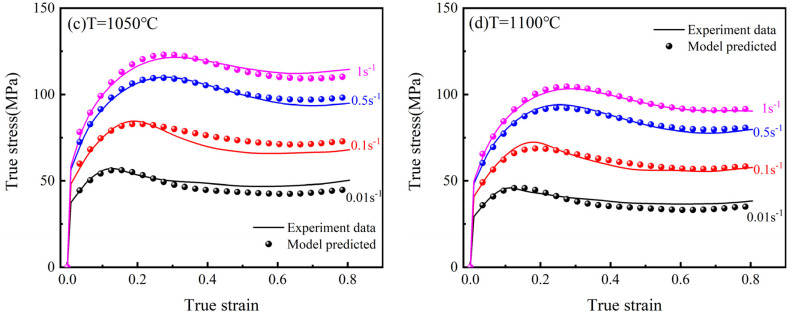
Arrhenius modeling results from the experimental data at different temperatures and strain rates. (**a**) T = 950 °C; (**b**) T = 1000 °C; (**c**) T = 1050 °C; (**d**) T = 1100 °C.

**Figure 12:**
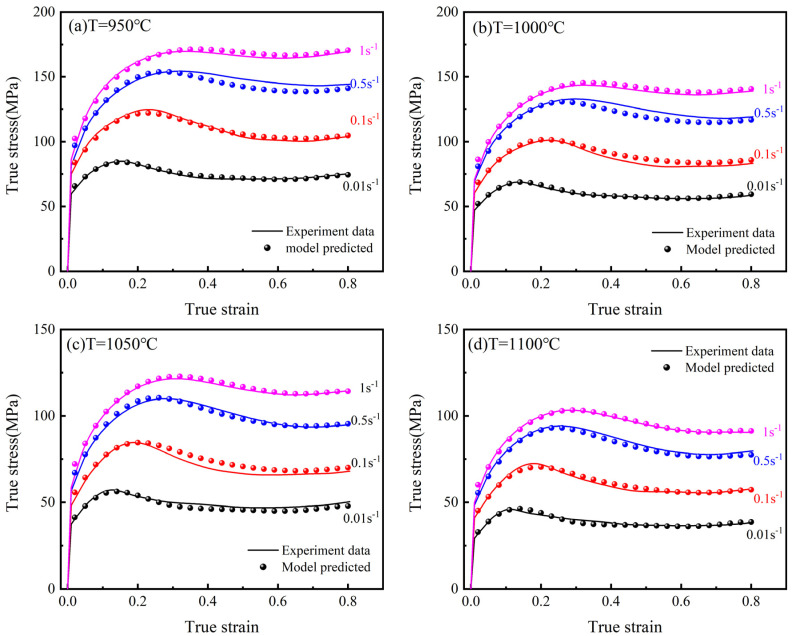
Modified Arrhenius modeling results from the experimental data at different temperatures and strain rates. (**a**) T = 950 °C; (**b**) T = 1000 °C; (**c**) T = 1050 °C; (**d**) T = 1100 °C.

The comparison of the experimental and the Arrhenius model predicted data is shown in Figure 11. When the strain rate is 0.01 s^−1^, the curves fit well at 950 °C and 1000 °C, with smaller deviations occurring at 1050 °C and 1100 °C. When the strain rate is 1 s^−1^, the curve fits well at 1100 °C and deviates more and more from the experimental data as the temperature decreases. This may be due to the fact that the model does not consider the coupling effect of temperature and strain rate, resulting in a large deviation between the predicted and experimental data under certain conditions. The experimental and the modified Arrhenius model predicted data are compared in Figure 12. It is clear that the modified Arrhenius model can accurately predict the hot compression deformation behavior of the 25CrMo4 steel.

In order to evaluate the accuracy of the constitutive models, the absolute relative error (ARE), the average absolute relative error (AARE), and the correlation coefficient (R^2^) are used as essential references. ARE, AARE, and R^2^ values can be calculated by:

(11) $$\mathrm{ARE}\left(\%\right)=\left|\frac{\sigma_{\exp}-\sigma_{\mathrm{CE}}}{\sigma_{\exp}}\right|\times 100\%$$

(12) $$\mathrm{AARE}\left(\%\right)=\frac{1}{N}\overset{N}{\underset{i=1}{\sum }}\left|\frac{\sigma_{\exp}-\sigma_{\mathrm{CE}}}{\sigma_{\exp}}\right|\times 100\%$$

(13) $$R^{2}=\frac{\sum_{i=1}^{N}\left(\sigma_{\exp}-{\bar{\sigma }}_{\exp}\right)\left(\sigma_{\mathrm{CE}}-{\bar{\sigma }}_{\mathrm{CE}}\right)}{\sqrt{\sum_{i=1}^{N}{\left(\sigma_{\exp}-{\bar{\sigma }}_{\exp}\right)}^{2}\sum_{i=1}^{N}{\left(\sigma_{\mathrm{CE}}-{\bar{\sigma }}_{\mathrm{CE}}\right)}^{2}}}$$

**Figure 13:**
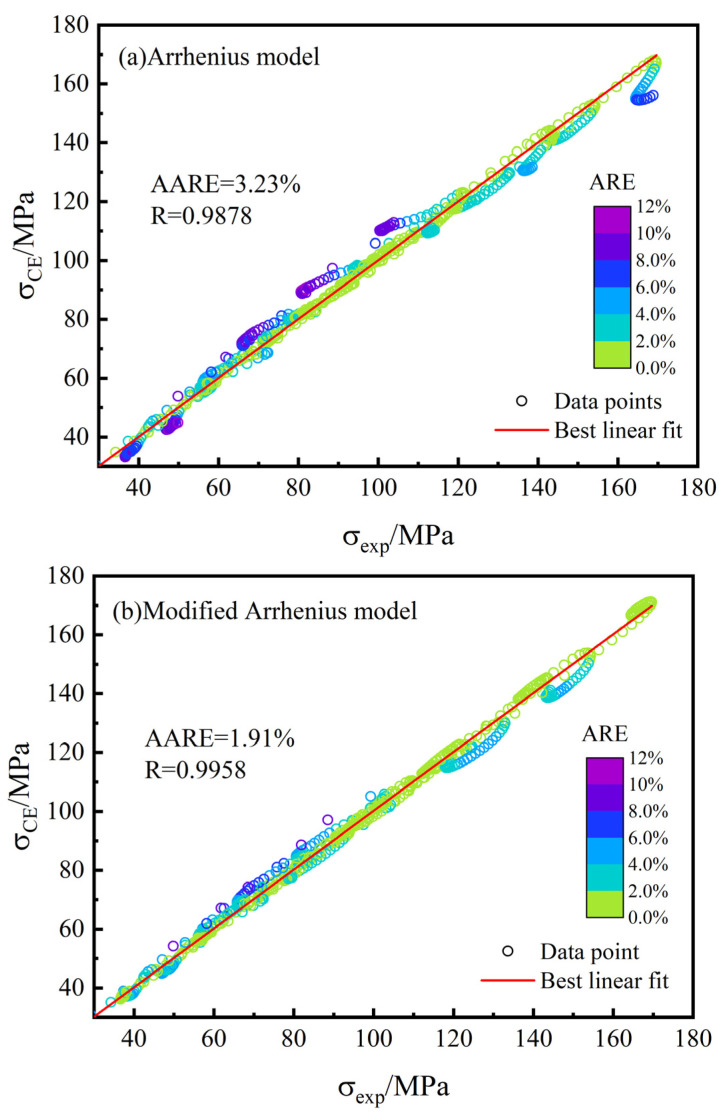
Correlation maps for constitutive models (**a**) Arrhenius model; (**b**) Modified Arrhenius model.

Figure 13 shows the correlation maps of the simulation curves of the constitutive model and the experimental results. The darker color of the points represents the larger ARE value. Compared with the original model, the AARE value of the modified Arrhenius model decreases from 3.23% to 1.91%, and the R^2^ value increases from 0.9878 to 0.9958. There are some data points in the Arrhenius model with ARE over 12% and below 8% overall, while in the modified Arrhenius model, almost no data points have ARE values above 8% and below 4% overall. This indicates that the modified Arrhenius model performs very well in describing the hot compression deformation behavior of the 25CrMo4 steel, and the predicted results are in general agreement with the experimental data.

## 5. Conclusions

In this study, isothermal compression and EBSD tests were used to investigate the hot deformation behavior of the 25CrMo4 steel at different temperatures and strain rates. The microstructure evolution and deformation mechanisms during hot deformation were analyzed. The strain-compensated Arrhenius model was calibrated, and a new modified Arrhenius model considering the coupling of temperature and strain rate was proposed. The conclusions are as follows:

(1) 25CrMo4 steel exhibits a significant softening effect at strain rates from 0.01 to 1 s^−1^ with a temperature range of 950–1100 °C during hot deformation, and the flow stress and critical strain increase with decreasing temperature and increasing strain rate.

(2) The occurrence of DRX is the main mechanism of the softening effect. As the temperature increases and the strain rate decreases, the dislocation density decreases, and the LAGB transforms to HAGB, promoting the formation and growth of recrystallized grains.

(3) The strain-compensated Arrhenius model is calibrated with the AARE value of 3.23% and the R^2^ value of 0.9878, with most of the ARE values less than 8%. At a temperature of 950 °C and strain rates of 0.1 and 1 s^−1^, the ARE values exceeded 12%.

(4) A modified Arrhenius model considering the coupling effect of temperature and strain rate is proposed, and compared with the original model, the AARE value is reduced to 1.91%, the R^2^ value is improved to 0.9958, and the overall ARE value is below 4%. Hence, the hot deformation behavior of the 25CrMo4 steel can be predicted accurately.

## Figures and Tables

**Table 1:** Chemical composition of the 25CrMo4 steel.

Composition	C	Si	Mn	Cr	Cu	Mo	Ni	V	Fe
wt.%	0.27	0.35	0.76	1.16	0.046	0.21	0.22	0.028	Bal.

**Table 2:** The coefficients of the Arrhenius model for the 25CrMo4 steel.

α	n	Q	lnA
$\alpha_{0}\;$= 0.0184	$n_{0}$ = 6.1621	$Q_{0}$ = 511.1411	$\ln A_{0}\;$= 43.9527
$\alpha_{1}$ = −0.1158	$n_{1}\;$= 16.8757	$Q_{1}$ = −1024.7073	$\ln A_{1}$ = −93.4998
$\alpha_{2}\;$= 0.7315	$n_{2}$ = −296.3931	$Q_{2}$ = −2723.1523	$\ln A_{2}$ = −263.5114
$\alpha_{3}$ = −2.3344	$n_{3\;}$= 1267.1775	$Q_{3}$ = 23,762.0810	$\ln A_{3}$ = 2291.8961
$\alpha_{4}\;$= 4.0344	$n_{4}$ = −2444.6079	$Q_{4}$ = −51,856.9870	$\ln A_{4}$ = −5058.5514
$\alpha_{5}$ = −3.5676	$n_{5}$ = 2245.9866	$Q_{5}\;$= 48,497.5153	$\ln A_{5}\;$= 4789.2922
$\alpha_{6}$ = 1.2572	$n_{6}$ = −798.4857	$Q_{6}$ = −16,925.7731	$\ln A_{6}$ = −1692.2681

**Table 3:** The coefficients of the modified Arrhenius model for the 25CrMo4 steel.

α	n	$m_{1}$	$m_{2}$	Q	lnA
$\alpha_{0}=$ 0.0184	$n_{0}=$ 6.0207	$m_{10}=$ 0.0017	$m_{20}=$ −3446	$Q_{0}=$ 511.1411	$\ln A_{0}=$ 43.9527
$\alpha_{1}=$ −0.1158	$n_{1}=$ 27.9892	$m_{11}=$ −0.0775	$m_{21}=$ 157,067	$Q_{1}=$ −1024.7073	$\ln A_{1}=$ −93.4998
$\alpha_{2}=$ 0.7315	$n_{2}=$ −479.4327	$m_{12}=$ 0.7093	$m_{22}=$ −1,560,050	$Q_{2}=$ −2723.1523	$\ln A_{2}=$ −263.5114
$\alpha_{3}=$ −2.3344	$n_{3}=$ 2227.0571	$m_{13}=$ −2.7677	$m_{23}=$ 646,9630	$Q_{3}=$ 23,762.0810	$\ln A_{3}=$ 2291.8961
$\alpha_{4}=$ 4.0344	$n_{4}=$ −4589.6447	$m_{14}=$ 5.1572	$m_{24}=$ −12,619,300	$Q_{4}=$ −51,856.9870	$\ln A_{4}=$ −5058.5514
$\alpha_{5}=$ −3.5676	$n_{5}=$ 4441.0982	$m_{15}=$ −4.6305	$m_{25}=$ 11,757,800	$Q_{5}=$ 48,497.5153	$\ln A_{5}=$ 4789.2922
$\alpha_{6}=$ 1.2572	$n_{6}=$ −1646.5750	$m_{16}=$ 1.6161	$m_{26}=$ −4,233,770	$Q_{6}=$ −16,925.7731	$\ln A_{6}=$ −1692.2681

**Figure A1:**
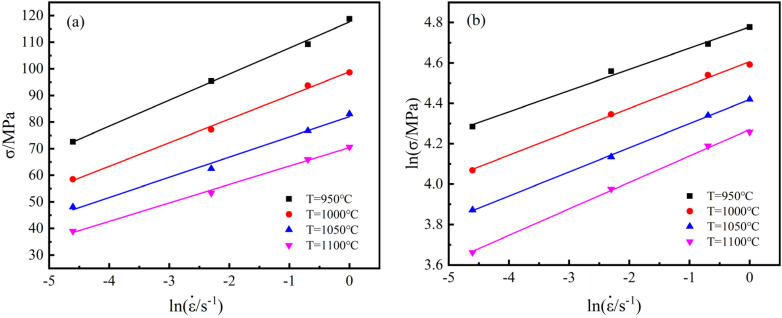
Fitting line (**a**) β by plotting $\ln\left(\dot{\varepsilon }\right)$ vs. σ; (**b**)$\;n_{1}$ by plotting $\ln\left(\dot{\varepsilon }\right)$ vs. ln(σ).

**Figure A2:**
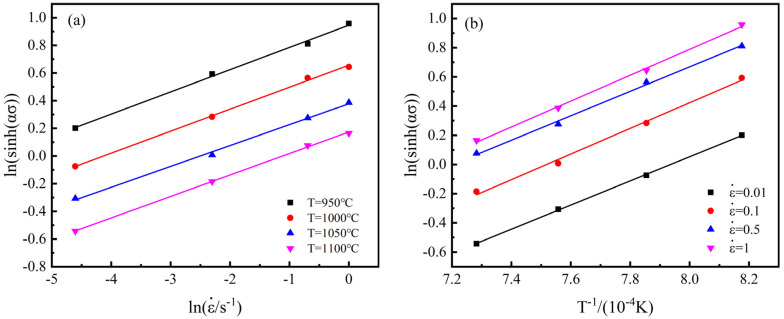
Fitting line (**a**) n by plotting $\ln\left(\dot{\varepsilon }\right)$ vs. ln[sinh(ασ)]; (**b**) Q/Rn by plotting ln[sinh(ασ)] vs. 1/T.

**Figure A3:**
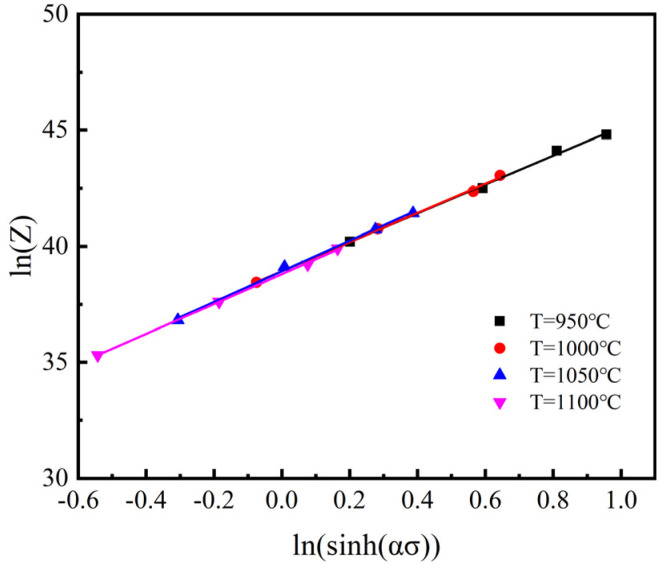
Fitting line, lnA by plotting lnZ vs. ln[sinh(ασ)].

**Figure A4:**
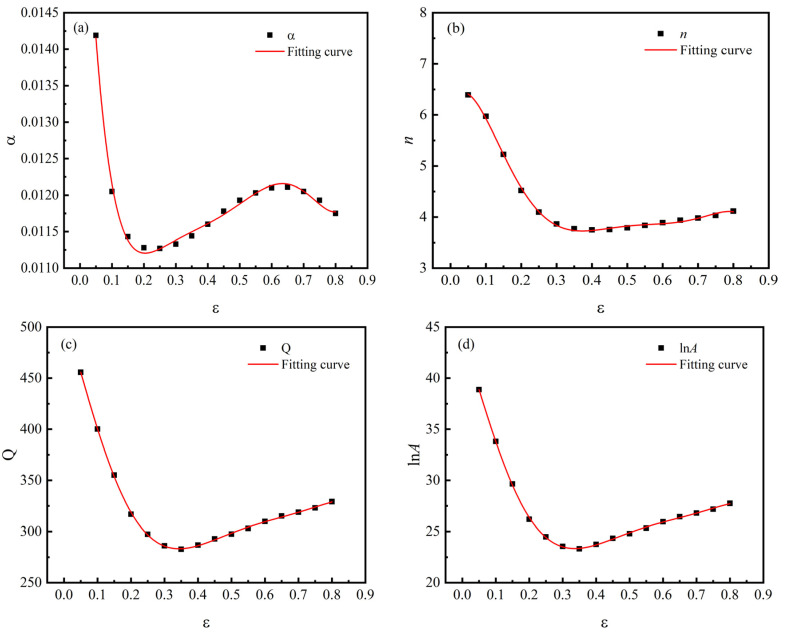
Variations of material constants with strain: (**a**) α; (**b**) n; (**c**) Q; (**d**) lnA.

## Nomenclature

**Symbol**	**Meaning**
α, β	material constant
ε	true strain
σ	true stress (MPa)
$\dot{\varepsilon }$	strain rate (s^−1^)
Q	thermal activation energy (KJ/mol)
T	deformation temperature (K)
R	molar gas constant (8.314 J/mol^−1^ K^−1^)
n, $n_{1}$	material constant
$m_{1}$,$\;m_{2}$	material constant
A	material constant
Z	Zener–Hollomon parameter
EBSD	electron backscatter diffraction
DRV	dynamic recovery
DRX	dynamic recrystallization
WH	work hardening
HAGB	high angle grain boundaries
LAGB	low angle grain boundaries
AARE	average absolute relative error
ARE	absolute relative error
R^2^	correlation coefficient

## Appendix A. Calibration of Parameters in the Arrhenius Model

### Appendix A.1. Determination of α Value

Taking the true strain of 0.05 as an example, the calculation process of the material parameters is as follows. For low and high stress levels, taking the logarithm of both sides of Equation (1), respectively:

(A1) $$\ln\left(\dot{\varepsilon }\right)=\ln\left(A\right)-\frac{Q}{\mathrm{RT}}+n_{1}\ln\left(\sigma \right)$$

(A2) $$\ln\left(\dot{\varepsilon }\right)=\ln\left(A\right)-\frac{Q}{\mathrm{RT}}+\beta \sigma$$

when T is kept constant, the values of $n_{1}$ and β are obtained from the slope of the fitting lines $\ln\left(\dot{\varepsilon }\right)$ vs. ln(σ) and $\ln\left(\dot{\varepsilon }\right)$ vs. σ shown in Figure A1, then taking the average values, $n_{1}\;$= 8.4868, β = 0.1205, and α = 0.0142 are obtained.

### Appendix A.2. Determination of n, Q, and A Values

For all the stress levels, Equation (1) can be represented as the following,

(A3) $$\dot{\varepsilon }=A\sinh{\left(\alpha \sigma \right)}^{n}\exp\left(-\frac{Q}{\mathrm{RT}}\right).$$

Taking the logarithm of both sides of the above Equation (A3):

(A4) $$\ln\left[\sinh\left(\alpha \sigma \right)\right]=\frac{\ln\left(\dot{\varepsilon }\right)}{n}+\frac{Q}{n\mathrm{RT}}-\frac{\ln A}{n}.$$

When T is kept constant, the partial derivatives of $\dot{\varepsilon }$ of Equation (A4) can be obtained:

(A5) $$n=\{{\frac{\partial \ln\left(\dot{\varepsilon }\right)}{\partial \ln\left[\sinh\left(\alpha \sigma \right)\right]}\}}_{T}.$$

When $\dot{\varepsilon }$ is kept constant, the partial derivatives of T of Equation (A4) can be obtained:

(A6) $$\frac{Q}{Rn}=\{{\frac{\partial \ln\left[\sinh\left(\alpha \sigma \right)\right]}{\partial \left(1/T\right)}\}}_{\dot{\varepsilon }}.$$

The values of n and Q/Rn are obtained from the slope of the fitting line $\ln\left(\dot{\varepsilon }\right)$ vs. ln[sinh(ασ)] and ln[sinh(ασ)] vs. 1/T shown in Figure A2, then taking the average values, n = 6.3918, Q/Rn = 8574.875, and Q = 455.7098 KJ/mol are obtained.

The effect of strain rate and temperature on flow characteristics can be expressed by the Zener–Hollomon (Z) parameter in the exponential equation [37]. The equation is as follows:

(A7) $$Z=\dot{\varepsilon }\exp\left(\frac{Q}{\mathrm{RT}}\right).$$

Taking the logarithm of both sides of the above Equation (A7):

(A8) $$\mathrm{lnZ}=\ln\dot{\varepsilon }+\frac{Q}{\mathrm{RT}}=\ln A+n\ln\left[\sinh\left(\alpha \sigma \right)\right].$$

The value of lnA is obtained from the intercept of the fitting line lnZ vs. ln[sinh(ασ)] in Figure A3, then taking the average value, lnA=38.8621 is obtained.

### Appendix A.3. Compensation of Strain

According to the above method, the material constants are computed under different deformation strains within the range of 0.05–0.8 and the interval of 0.05. The relationships between material constants (α,n,Q, and lnA) and strain are shown in Figure A4.
